# [^18^F]Fluorocholine PET/CT in pediatric primary hyperparathyroidism

**DOI:** 10.1007/s00247-024-06036-x

**Published:** 2024-08-31

**Authors:** Giorgio Treglia, Benedetto Zanetti, Giacomo D. Simonetti

**Affiliations:** 1 Imaging Institute of Southern Switzerland, Ente Ospedaliero Cantonale, via Gallino 12, CH-6500, Bellinzona, Switzerland; 2 Università della Svizzera Italiana, Lugano, Switzerland; 3 Pediatric Institute of Southern Switzerland, Ente Ospedaliero Cantonale, Bellinzona, Switzerland


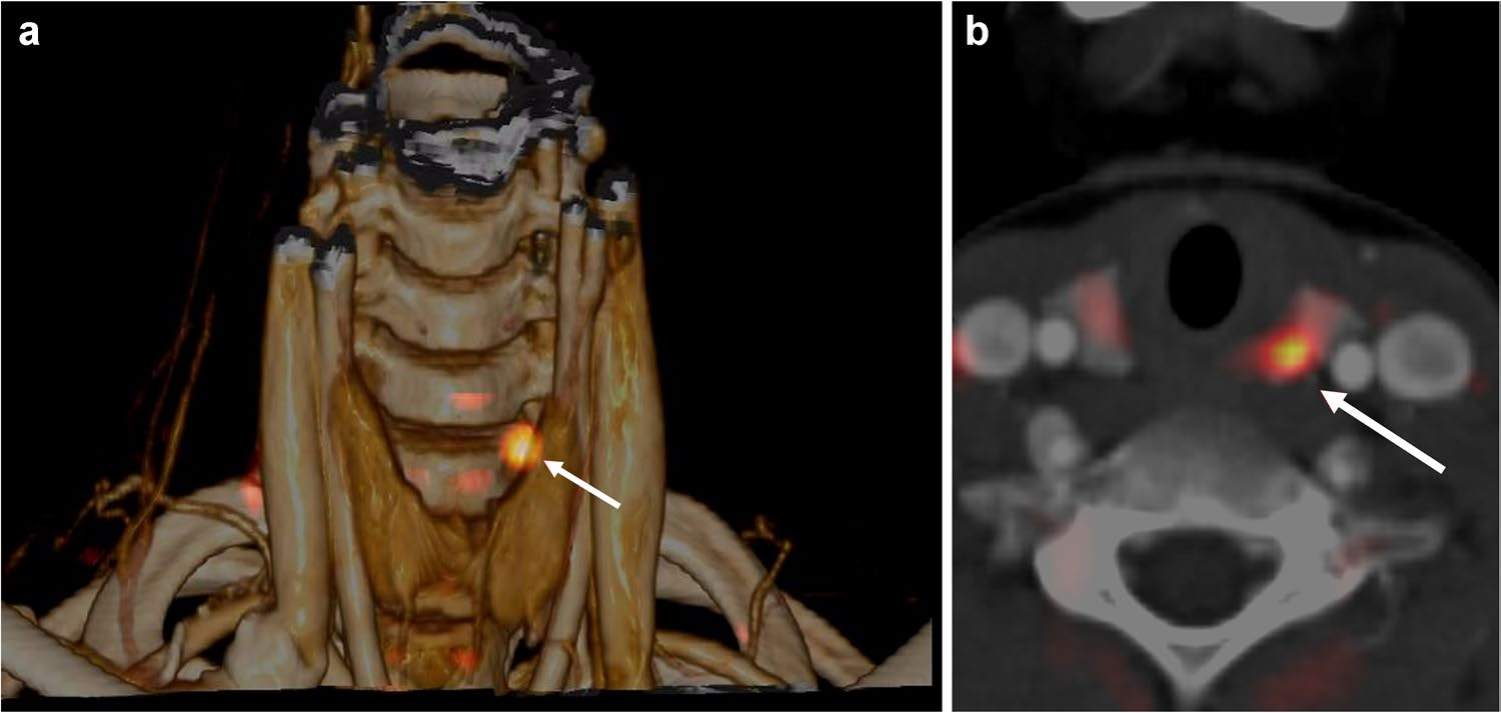


An 11-year-old male patient with diagnosis of primary hyperparathyroidism and history of kidney stones underwent [^18^F]fluorocholine positron emission tomography/computed tomography (PET/CT) to localize hyperfunctioning parathyroid gland(s) after negative or inconclusive first-line imaging methods (ultrasound and parathyroid scintigraphy using SPECT/CT acquisition). PET/CT was performed 45 min after the injection of 74 MBq of [^18^F]fluorocholine (field-of-view, 50 cm). Volume-rendered (**a**) and axial (**b**) [^18^F]fluorocholine PET/CT images detected an area of increased radiotracer uptake corresponding to a small nodule (maximum diameter, 8 mm) located behind the upper portion of the left thyroid lobe (*arrows*) highly suspicious for eutopic left superior parathyroid adenoma. Based on these PET/CT findings, the patient underwent left parathyroidectomy with excision of a pathology proven parathyroid adenoma. Primary hyperparathyroidism is a rare condition in children and treatment usually includes surgical removal of hyperfunctioning parathyroid gland(s). The figure demonstrates that [^18^F]fluorocholine PET/CT may be an effective imaging method to detect and localize hyperfunctioning parathyroid glands in pediatric patients with primary hyperparathyroidism.

## Data Availability

No datasets were generated or analysed during the current study.

